# Correlations of Vascular Architecture and Angiogenesis with Pituitary Adenoma Histotype

**DOI:** 10.1155/2014/989574

**Published:** 2014-11-09

**Authors:** Shingo Takano, Hiroyoshi Akutsu, Takuma Hara, Tetsuya Yamamoto, Akira Matsumura

**Affiliations:** Department of Neurosurgery, Faculty of Medicine, University of Tsukuba, 1-1-1 Tennodai, Tsukuba, Ibaraki 305-8575, Japan

## Abstract

Vascular endothelial growth factor (VEGF) is a potent angiogenic factor in solid tumors. However, its role in angiogenesis in pituitary adenoma is controversial. Angiogenesis in solid tumors including pituitary adenoma is commonly evaluated by microvascular density (MVD). Here, we evaluated MVD and the role of VEGF in vascular architecture in 51 pituitary adenomas (24 nonfunctioning, 13 prolactin-secreting, 10 growth hormone-secreting, 3 adrenocorticotropic hormone-secreting, and 1 thyroid-stimulating hormone-secreting). Paraffin sections were stained with CD34 and VEGF. MVD and vascular architecture parameters (vessel area, diameter, perimeter, and roundness) were evaluated in CD34-stained sections. Immunohistochemistry showed 27/51 tumors (53%) were VEGF-positive. There were no significant differences in MVD, any vascular parameter, or adenoma volume between VEGF-positive and VEGF-negative tumors. VEGF mRNA expression was significantly higher in VEGF-positive tumors. There were no significant correlations between VEGF mRNA expression and MVD or vascular parameters. However, vessel diameter and perimeter were significantly larger in prolactin-secreting than nonfunctioning and growth hormone-secreting macroadenomas. The difference in vessel diameter was observed among both VEGF-positive and all adenomas (micro- and macroadenoma). Thus, VEGF may have limited roles in the development of vascular architecture and tumor angiogenesis in pituitary adenomas, but the differences in vessel architecture by histotype (i.e., larger vessel diameter and perimeter in prolactin-secreting adenomas) suggest the hormonal regulation of vessel architecture rather than angiogenesis

## 1. Introduction

Angiogenesis, a fundamental process in tumor growth and development, is less prominent in adenomas than normal pituitary tissue [1–3]. The behavior of angiogenesis as a function of hormonal secretion and other characteristics of pituitary tumors remain controversial [4–8]. Vascular endothelial growth factor (VEGF) is a potent angiogenic factor expressed in many solid tumors [9]. It is considered an important biomarker in pituitary neoplasms [10]. VEGF was discovered in the conditioned medium of pituitary follicular cells in 1989 [11] and is still focused there as of 2012 [12]. Moreover, VEGF may be an important humoral factor for both normal and tumorous pituitary tissues.

The degree of angiogenesis in solid tumors including pituitary adenomas is commonly evaluated by microvascular density (MVD), in which the number of vessels in a given area is counted. However, angiogenesis possesses many aspects of neovascularization, such as vessel number, branching pattern, diameter, and shape. Among pituitary hormones, prolactin (PRL) and adrenocorticotropic hormone (ACTH) are reported to be related to vascular development and endothelial cell function [13–15]. Therefore, these hormone-secreting adenomas may have different vasculature and angiogenic characteristics compared to other types of adenomas.

Therefore, this study determined the roles of VEGF in angiogenesis and vascular architecture in pituitary adenomas. In addition, the differences in vascular architecture parameters other than MVD were determined between different histotypes of pituitary adenomas.

## 2. Materials and Methods

### 2.1. Pituitary Adenoma Samples

A total of 51 pituitary adenomas (24 nonfunctioning, 13 PRL-secreting, 10 growth hormone- (GH-) secreting, 3 ACTH-secreting, and 1 thyroid-stimulating hormone- (TSH-) secreting) obtained during surgery were fixed in formalin, embedded in paraffin, and sectioned at 5 *μ*M. The adenoma volume was measured by magnetic resonance imaging according to the 3-dimensional diameter (AP: maximum diameter of the anterior-to-posterior direction in a sagittal section; LR: maximum diameter of the left-to-right direction in a coronal section; up and down: maximum diameter of the up-to-down direction in a coronal or sagittal section). The cystic component of the adenoma was measured separately and not included in the calculation. Macroadenomas (i.e., >10 mm in diameter in one dimension) were observed in 24 of 24 nonfunctioning, 10 of 13 PRL-secreting, 8 of 10 GH-secreting, 2 of 3 ACTH-secreting, and 0 of 1 TSH-secreting adenomas. For functioning adenomas, preoperative serum hormone levels (i.e., GH, PRL, and ACTH) were determined.

### 2.2. CD34 and VEGF Immunohistochemistry and Measurement of Vascular Architecture

**Figure 1:**
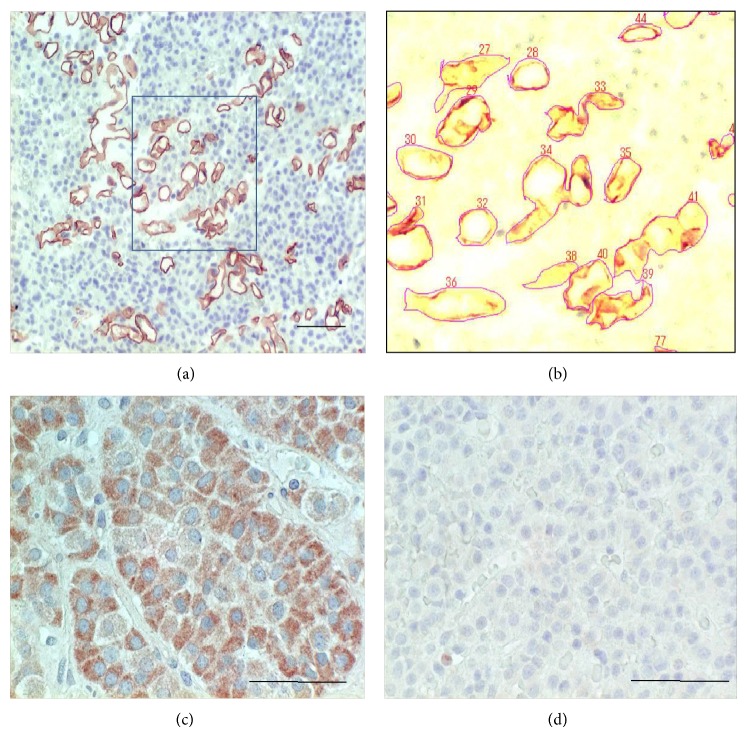
Determination of microvascular density and vascular architecture (a, b) and VEGF immunohistochemistry (c, d). CD34-stained fields (1.0 mm^2^) (a) are input into the image analyzer, and each vessel contour is manually traced in order to measure vessel density (number), vessel area (%), vessel diameter (*μ*m), vessel perimeter (*μ*m), and vessel roundness (0-1) (b). The area of the black box in (a) is magnified to (b). VEGF immunohistochemistry: positive tumor with clear cytoplasmic staining (c) and negative tumor (d). Original magnification (a): 200x, (c), (d): 400x. Bar 100 *μ*m.

Paraffin sections were stained with monoclonal anti-CD34 antibody (1 : 100, M7165; Dako) and polyclonal anti-VEGF antibody (1 : 100, A20; Santa Cruz) by the streptavidin-biotin-peroxidase method (Dako LSAB2 System) as described previously [16]. The same concentrations of chromatographically purified mouse IgG and rabbit IgG (Dako) were used as negative controls. CD34-stained sections were evaluated for MVD, and vascular architecture parameters including vessel area, diameter, perimeter, and roundness were analyzed by using an image analyzer system (WinROOF, Mitani Corporation, Japan); WinROOF is an integrated software system for image processing, measurement, and data processing to support all image analysis-related operations [17, 18]. The defined area for the measurement of these parameters was 1.0 mm^2^. CD34-stained fields of 1.0 mm^2^ (Figure 1(a)) were input into the image analyzer, and each vessel contour was manually traced in order to measure vessel density (number), vessel area (%), vessel diameter (*μ*m), vessel perimeter (*μ*m), and vessel roundness (0-1; 1 = completely round) (Figure 1(b)). Three different fields in each case were measured, and the median values were used for analysis. VEGF immunohistochemistry was defined as positive if more than 10% of adenoma cells were positive.

### 2.3. VEGF mRNA Expression by RT-PCR

Among the 51 pituitary adenomas, frozen tissues of 19 adenomas (12 nonfunctioning, 4 PRL-secreting, 2 GH-secreting, and 1 ACTH-secreting) were available. Total RNA was extracted, and VEGF mRNA expression was measured by RT-PCR as described previously [19]. Briefly, 1 *μ*g total RNA was reverse-transcribed by MuLV reverse transcriptase in the presence of random hexamers followed by the indicated cycles of PCR (95°C for 1 min, 55°C for 1 min, and 72°C for 1 min) in the presence of 2 *μ*MVEGF-specific primers (28 cycles) or *β*-actin-specific primers (16 cycles) as a control. The VEGF primers included the reverse primer (5′-CCTGGTGAGAGATCTGGTTC-3′) spanning bases 861–842 and the forward primer (5′-TCGGGCCTCCGAAACCATGA-3′) spanning bases 141–160. The *β*-actin primers included the reverse primer (5′-GGAGTTGAAGGTAGTTTCGTG-3′) spanning bases 2429–2409 and the forward primer (5′-CGGGAAATCGTGCGTGACAT-3′) spanning bases 2107–2126. The VEGF primers were chosen because they amplified exons 3–8, enabling us to distinguish VEGF splicing variants. PCR products of 516 and 648 bp corresponded with VEGF_121_ and VEGF_165_, respectively. RT-PCR products were quantified by densitometry.

### 2.4. Statistical Analysis

Vascular density, tumor volume, the densitometric values of VEGF and *β*-actin, and adenoma architecture parameters (i.e., area, diameter, perimeter, and roundness) are expressed as mean ± SD. The one-way ANOVA with post hoc Tukey's comparisons was used for multiple groups, while the *t*-test was used for comparisons of 2 groups. Pearson's correlation coefficients (*r*) were also calculated. All *P* values are two-sided, and the level of significance was set at *P* < 0.05.

## 3. Results

### 3.1. VEGF Expression and Pituitary Adenoma

**Figure 2:**
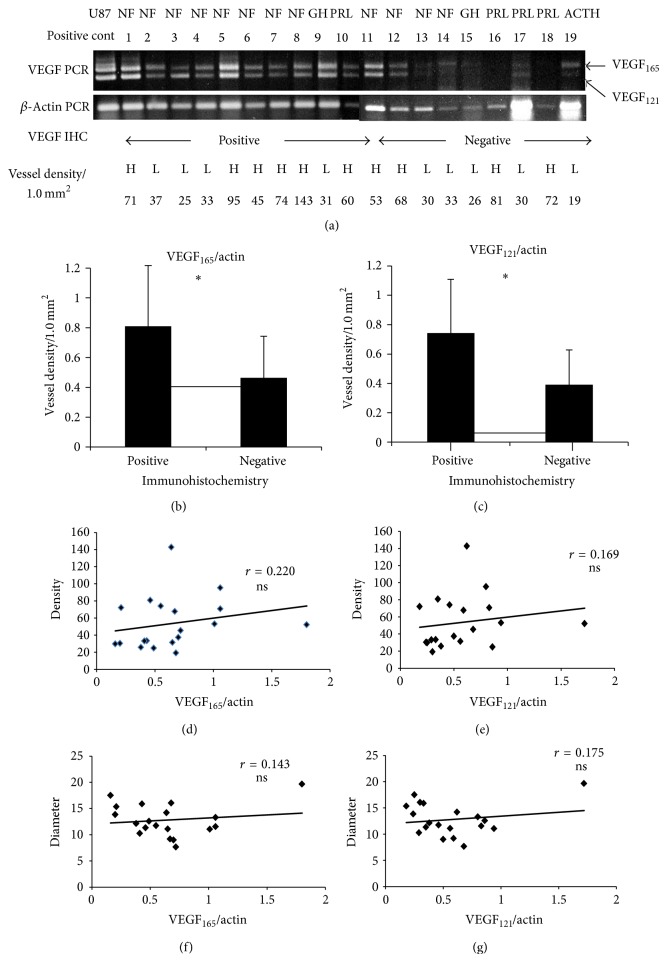
Determination of VEGF mRNA expression and immunohistochemistry. (a) VEGF mRNA expression and corresponding VEGF immunohistochemistry results (positive and negative) and vessel density (number/0.13 mm^2^, *H* ≥ 40, *L* < 40). (b) The VEGF_165_/*β*-actin ratio was significantly higher in VEGF-positive group than the VEGF-negative group (*P* < 0.05). (c) The VEGF_121_/*β*-actin ratio was significantly higher in the VEGF-positive group than the VEGF-negative group (*P* < 0.05). (d) The correlation between VEGF_165_/*β*-actin ratio and vessel density was not significant (*r* = 0.22). (e) Correlation between VEGF_121_/*β*-actin ratio and vessel density is not significant (*r* = 0.169). (f) Correlation between VEGF_165_/*β*-actin ratio and vessel diameter is not significant (*r* = 0.143). (g) Correlation between VEGF_121_/*β*-actin ratio and vessel density is not significant (*r* = 0.175).

Immunohistochemistry showed 27/51 tumors (53%) were positive for VEGF (Figures 1(c) and 1(d)). There were no significant differences between the VEGF-positive and VEGF-negative groups with respect to MVD, any vascular architecture parameter, or adenoma volume (Table 1). In 19 tumors, VEGF mRNA and protein expressions were measured simultaneously (Figure 2(a)). The VEGF_165_/actin ratio was significantly higher in VEGF-positive tumors (0.81 ± 0.91) than negative tumors (0.46 ± 0.23) (*P* = 0.045, Figure 2(b)). Likewise, the VEGF_121_/actin ratio was significantly higher in VEGF-positive tumors (0.74 ± 0.37) than negative tumors (0.39 ± 0.24) (*P* = 0.019, Figure 2(c)). There was no significant correlation between VEGF_165_ or VEGF_121_ mRNA expression and MVD or any vascular architecture parameter (Table 2, Figures 2(d), 2(e), 2(f), and 2(g)). Thus, these results indicate VEGF has only small roles in the vascular architecture and angiogenesis of pituitary adenomas.

### 3.2. Vascular Architecture in Different Histotypes of Pituitary Adenomas

**Figure 3:**
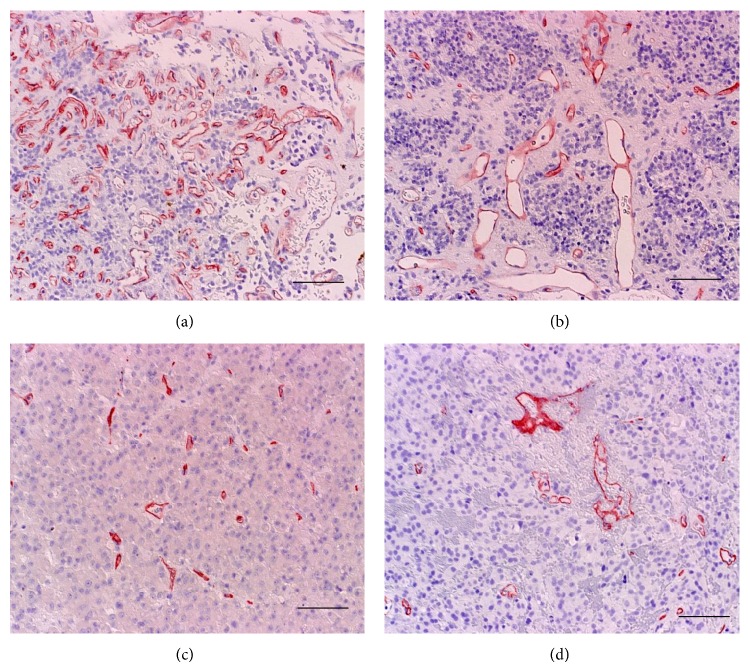
Representative vascular architecture (CD34 stain) of nonfunctioning (a), prolactin-secreting (b), GH-secreting (c), and ACTH-secreting (d) macroadenomas. Vessel diameter is larger in prolactin- and ACTH-secreting adenomas than nonfunctioning and GH-secreting adenomas. Vessel density and area are lower in ACTH-secreting adenoma than nonfunctioning and PRL-secreting adenomas. Original magnification: 200x. Bar 100 *μ*m.

**Figure 4:**
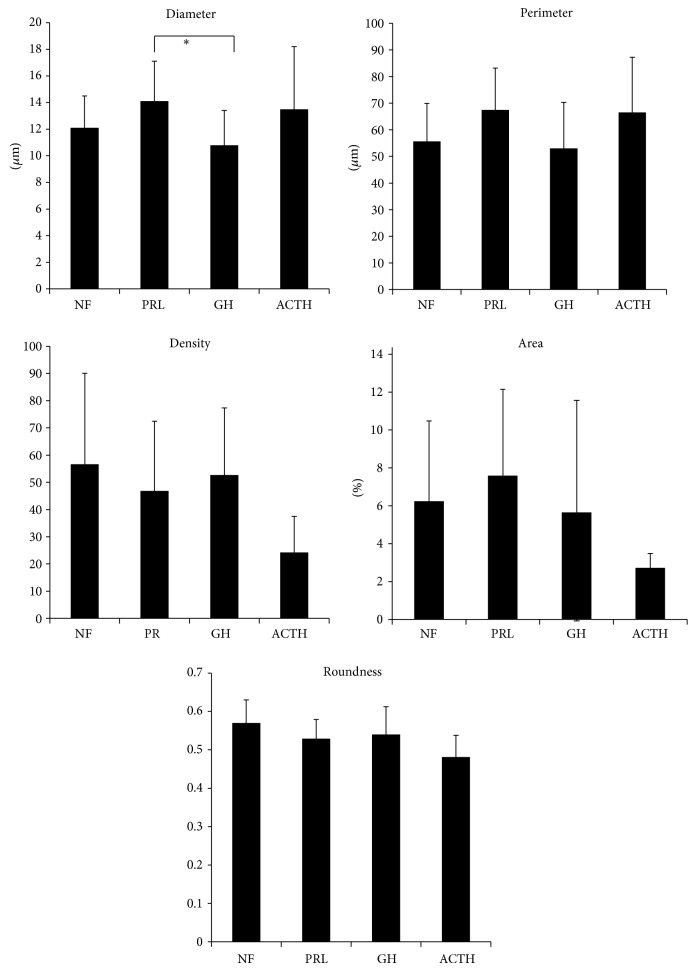
Differences in the vascular architecture of all types of pituitary adenomas. Vessel diameter is significantly larger in prolactin-secreting adenomas than GH-secreting adenomas. Vessel density, area, and roundness are lower tendency in ACTH-secreting adenoma than nonfunctioning adenomas. ^*^ *P* < 0.05.

**Figure 5:**
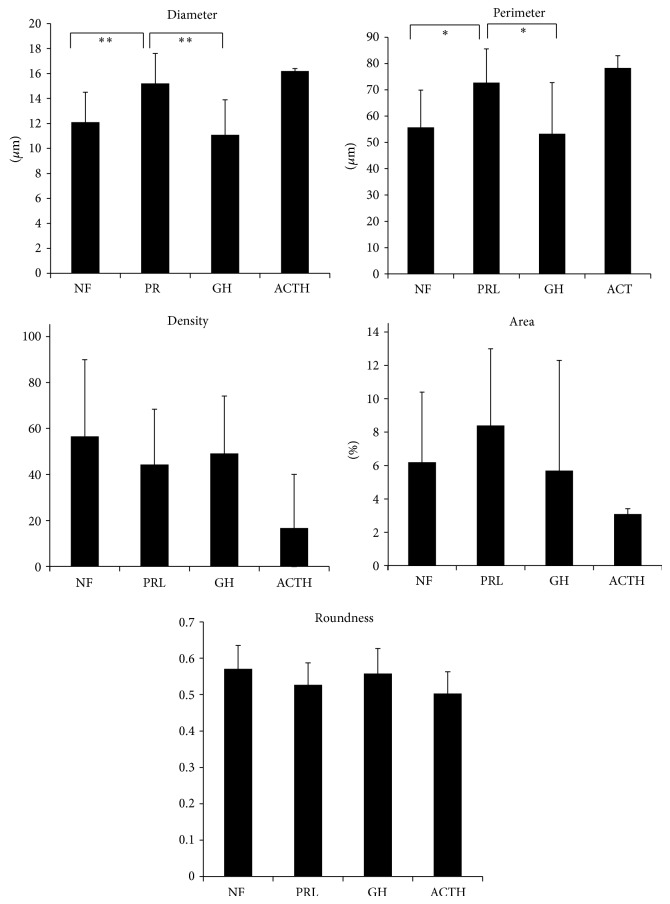
Differences of vascular architecture in macroadenoma. Vessel diameter and perimeter are significantly larger in prolactin-secreting adenomas than nonfunctioning and GH-secreting adenomas. ^*^ *P* < 0.05; ^**^ *P* < 0.01.

The vascular architecture parameters in different histotypes of adenomas are shown in Table 3 and Figure 3. Vessel diameter was significantly larger in PRL-secreting adenomas than GH-secreting adenomas (Figure 4). Meanwhile, vessel density and area tended to be lower in ACTH-secreting adenomas than nonfunctioning adenomas, although the differences were not significant. Because VEGF may influence vascular architecture, VEGF-positive adenomas alone (*n* = 27) were analyzed. Again, vessel diameter was significantly larger in PRL-secreting adenomas than GH-secreting adenomas. Because adenoma volume may influence vascular architecture, macroadenomas alone (*n* = 44) were analyzed. PRL-secreting adenomas had significantly larger perimeter and vessel diameter than nonfunctioning and GH-secreting adenomas (Figure 5). In summary, PRL-secreting adenomas, especially macroadenomas, have larger diameters and perimeters than nonfunctioning and GH-secreting adenomas.

**Figure 6:**
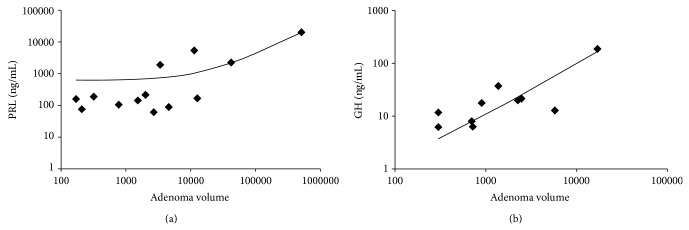
Correlation between adenoma volume and hormone value. (a) Prolactin-secreting adenomas. (b) GH-secreting adenomas.

In PRL-secreting adenomas, serum PRL level was strongly correlated with adenoma volume (*r* = 0.9679, *P* < 0.001, Figure 6(a)). However, adenoma volume was not correlated with other vessel architecture parameters (density: *r* = 0.2008, *P* = 0.5313; area: *r* = 0.3315, *P* = 0.2925; diameter: *r* = 0.162, *P* = 0.6146; perimeter: *r* = 0.138, *P* = 0.6685; roundness: *r* = −0.168, *P* = 0.6014). Furthermore, in GH-secreting adenomas, serum GH level was strongly correlated with adenoma volume (*r* = 0.9412, *P* < 0.001, Figure 6(b)).

## 4. Discussion

Vascular architecture parameters differed among adenoma histotypes. In macroadenomas, PRL-secreting adenomas had larger vessel diameter and perimeter than nonfunctioning and GH-secreting adenomas. In all adenomas (including macro- and microadenomas) and VEGF-positive adenomas, vessel diameter in PRL-secreting adenomas was larger than that in GH-secreting adenomas. In addition, VEGF expression did not reveal any differences in MVD, vascular architecture, or tumor volume among adenoma histotypes. Thus, VEGF expression in pituitary adenoma has little effect on angiogenesis, vascular architecture, or histotype.

### 4.1. Role of VEGF in Pituitary Adenomas

VEGF is a potent angiogenic factor for solid tumors. The global standard for the measurement of tumor and physiological angiogenesis is immunohistochemical counting of vessels per defined hotspot area, that is, MVD, owing to its simplicity [20]. Therefore, angiogenesis of pituitary adenomas has been reported in many recent studies evaluating VEGF protein expression (measured by immunohistochemistry and western blot analysis), VEGF mRNA expression, and MVD. Nevertheless, the role of VEGF in angiogenesis in pituitary adenoma remains inconclusive. VEGF is reported to be a potent angiogenic factor in pituitary adenomas [4]. On the contrary, VEGF is found equally in normal tissue and adenomas and among tumors of different histotype [3]. Pituitary tumor cells are capable of producing VEGF, which may be involved in tumor angiogenesis [21]. Furthermore, VEGF mRNA and protein are expressed in all pituitary adenomas [22]. Thus, these findings suggest VEGF may not be a potent angiogenic factor in pituitary adenomas.

One of the reasons for these controversial results is the limitation of immunohistochemistry, which is semiquantitative. Also, MVD is only one aspect of neovascularization. Therefore, as an alternative, the present study evaluated angiogenesis by immunohistochemistry as well as mRNA expression to evaluate VEGF expression and vascular architecture parameters, and MVD.

A recent study demonstrates that VEGF mRNA expression differs among histological subtypes. The extension on magnetic resonance imaging indicates VEGF expression is related to suprasellar extension, being expressed more on tumors with extrasellar growth than intrasellar growth [23]. However, no relationship between VEGF expression and MVD was found in the present study. Hence, VEGF may be related to tumor growth (e.g., inhibition of apoptosis) but not tumor angiogenesis.

In the present study, high VEGF protein and mRNA expression did not reflect MVD, vascular architecture, tumor volume, or any particular histotype, suggesting VEGF plays little role in pituitary adenoma angiogenesis and growth. Stromal cell-derived factor-1 (SDF-1) is reported to be related to MVD in pituitary adenoma as a CD34-positive endothelial progenitor cell homing factor [24]. Hence, studies investigating the regulation of this novel angiogenic factor and VEGF in the angiogenesis of pituitary adenomas are warranted.

### 4.2. Vascular Architecture

MVD is one of the most widely used estimators of tumor microvascularity in two-dimensional histological sections [5, 25]. However, MVD has several substantial limitations mainly owing to the complex biology of tumor microvasculature [26] and the irregular geometry that microvascular systems assume in real space [27]. Until now, the finding that MVD in the normal anterior pituitary was significantly higher than that in tumors was generally accepted [3, 7]. However, the differences in MVD among adenoma histotypes are highly discordant in the literature. Adenomas with higher MVD are thyrotroph cell adenomas, while those with lower MVD are PRL cell adenomas [6]. Micro- and macroadenomas that secrete GH or ACTH have comparable vascular densities, whereas macroprolactinomas are significantly more vascular than microprolactinomas [8].

Several methods for quantitatively analyzing pituitary adenoma microvasculature besides MVD have been applied. Fractal analysis is emerging as a potential effective model for this aim [1]. In addition, the combination of different types of immunostaining techniques such as CD105 (Endoglin) [28, 29] and Endocan [30] for the detection of microvessels in pituitary adenomas by using fractal analysis is an objective computer-aided technique for quantifying and describing the morphological aspects of microvessels that has potential implications in future clinical and surgical applications [2].

Geometrically, human vascularity is a complex three-dimensional system; its sizes, shapes, and connecting patterns are highly variable in two-dimensional histological sections. This geometrical complexity is the main cause of discordant results when assessing microvascularity in surgical tissue specimens [1].

In 2003, Vidal et al. suggested using microvascular structural entropy as a new index for the simultaneous measurement of the size of vessels and their arrangements in two-dimensional sections of pituitary tumors [31]. They found that microvascular structural entropy is significantly higher in pituitary adenomas than PRL-secreting carcinomas. In 2007, Di leva et al. first estimated the global complexity of the two-dimensional microvasculature of normal pituitary glands and pituitary adenomas by quantifying their box counting fractal dimension [1]. They found that the microvasculature of normal pituitary specimens is significantly more geometrically complex than that of pituitary adenomas. On the basis of the principles of fractal geometry, this indicates normal pituitary tissue is more vascularized than pituitary adenomas and that the microvasculature of normal pituitary glands is more complex than that of pituitary adenomas. These studies collectively highlight the importance of using geometrical vascular architectural elements other than MVD to evaluate the complex form of tumor vascularity.

However, these geometrical analyses require complex calculations. MVD is only one functional aspect of a tumor microvascular bed; other aspects such as morphology (i.e., tortuosity, branching pattern, and microvessel diameter), maturation, and endothelial wall permeability represent equally important attributes. Among these vessel architecture parameters, we chose simple parameters—vessel diameter, perimeter, and roundness—in addition to MVD. The results demonstrate the importance of vessel diameter and perimeter as biomarkers of different histotypes; PRL-secreting adenomas had larger vessel diameter and perimeter than nonfunctioning and GH-secreting adenomas.

### 4.3. Significance of Larger Vessel Diameter and Perimeter in PRL-Secreting Adenomas

With regard to PRL-secreting adenomas, vascularity evaluated by MVD is reported to be related to pretreatment hormone production, invasiveness, and surgical cure with lower vascularity [8]. However, whether PRL also influences endothelial cells and whether there are functional consequences of PRL-induced signaling from the perspective of angiogenesis remains elusive. PRL directly stimulates endothelial cell migration and tube formation both in vitro and in vivo in chorioallantoic membrane [32]. In the present study, serum PRL level was strongly correlated with adenoma volume as described previously. However, serum PRL level did not reflect specific vascular architecture parameters. Hyperprolactinemia with PRL-secreting adenomas may have some effect on tumor vasculature.

Blood vessels are emerging as important PRL targets, contributing to PRL's hormonal functions. PRL promotes angiogenesis and is proteolytically cleaved into vasoinhibins, a family of peptides (including 16 kDa PRL) with potent antiangiogenic and blood vessel regression effects [33, 34]; 16 kDa PRL impairs functional tumor neovascularization by inhibiting vessel maturation and for the first time demonstrated that an endogenous antiangiogenic agent disturbs notch signaling [35]. Recent results suggest tissue enzymes play an important role in the production of this form of PRL in several tissues including the retina, myocardium, and mammary glands. The cleavage leading to the production of 16 kDa PRL may occur extracellularly in the interstitial medium and therefore in the vicinity of blood capillaries [36].

Like PRL-secreting adenomas, circulating levels of PRL are elevated in diabetes; accordingly, they are higher in diabetes patients without retinopathy than in those with proliferative diabetic retinopathy, which is an angiogenic disease. Circulating PRL influences the progression of diabetic retinopathy after its intraocular conversion to vasoinhibins. Therefore, inducing hyperprolactinemia may represent a novel treatment strategy against diabetic retinopathy [37].

The synthesis of antiangiogenic factors by tumor cells has been demonstrated. Actively growing primary tumors can secrete antiangiogenic factors into the circulation as is the case for angiostatin and endostatin, which can maintain tumors in a dormant state [38, 39]. It remains unknown if PRL-secreting adenomas can produce antiangiogenic factors such as 16 kDa PRL. PRL and/or its inhibitor may directly influence adenoma vasculature. In gliomas treated with bevacizumab, an anti-VEGF antibody, tumor vasculature becomes dilated and thin, which suggests normalization, compared to that in nontreated tissue in experimental animals [40] and humans [41, 42]. In contrast, in human corneal neovascularization, topical bevacizumab decreases corneal vessel diameter [43]. Meanwhile, the effect of somatostatin analog treatment on vessel diameter in PRL-secreting adenomas would be interesting to investigate in the future.

### 4.4. Significance of Vessel Architecture in ACTH-Secreting Adenomas

There are few studies concerning ACTH-secreting adenomas, vascularity, and/or vascular architecture. Among 46 adenomas (18 nonfunctioning, 12 ACTH-secreting, 12 GH-secreting, and 4 PRL-secreting), there was no difference among histotypes with respect to MVD [3]. In another report of 112 (30 GH-secreting, 25 PRL-secreting, 15 ACTH-secreting, and 42 nonfunctioning tumors) and 13 normal anterior pituitary gland specimens, ACTH-secreting adenomas were, like microprolactinomas, had much lower vascular density than the normal pituitary tissue and other secreting and nonsecreting tumor types [7]. Micro- and macroadenomas that secrete ACTH have comparable vascular densities [8]. In the present study, ACTH-secreting adenomas tended to have larger vessel diameter and perimeter and lower density than nonfunctioning and GH-secreting adenomas.

The direct action of ACTH on vessels has not been reported. However, the direct actions of cortisol on endothelial cells and vascular permeability have been demonstrated. Interestingly, glucocorticoids directly interact with glucocorticoid receptors on vascular endothelial cells to inhibit tube-like formation. This action is due to alterations in cell morphology rather than the inhibition of endothelial cell viability, migration, or proliferation and may be mediated in part by the induction of thrombospondin-1 [44]. Therefore, ACTH-secreting adenomas may alter vascular architecture.

## 5. Conclusion

VEGF expression plays little role in angiogenesis in pituitary adenomas. Taken together the fact that pituitary adenomas are hypovascular compared to the normal pituitary gland, in biological terms, we can speculate that pituitary adenomas may progress via a nonangiogenic and VEGF-independent pathway. The differences in vessel architecture in different histotypes, particularly larger vessel diameter and perimeter in PRL-secreting adenomas than nonfunctioning and GH-secreting adenomas, suggest the hormonal regulation of vessel architecture other than angiogenesis.

## Figures and Tables

**Table 1:** 

	VEGF pos		VEGF-negative		Statistics
	mean	SD	mean	SD	
All cases	*n* 27		*n* 24		
Density/1.0 mm^2^	57.2	30.6	46.0	27.80	ns
Area %	7.17	5.54	5.24	2.89	ns
Diameter *μ*m	12.3	0.6	12.5	0.56	ns
Perimeter *μ*m	58.9	18.4	59.4	13.40	ns
Roundness	0.55	0.06	0.55	0.08	ns
Tumor volume mm^3^	28035.7	97661.7	4342	4500.9	ns

NF	*n* 11		*n* 13		
Density/1.0 mm^2^	63.8	11.1	50.6	8.50	ns
Area %	7.13	5.37	5.49	3.00	ns
Diameter *μ*m	11.9	2.65	12.2	2.30	ns
Perimeter *μ*m	56.4	17.4	55.1	11.40	ns
Roundness	0.56	0.03	0.58	0.08	ns
Tumor volume mm^3^	13519.1	16887.9	6648.1	4654.6	ns

PRL	*n* 7		*n* 6		
Density/1.0 mm^2^	50.3	9.8	42.6	11.10	ns
Area %	9.19	5.18	5.73	3.12	ns
Diameter *μ*m	14.9	2.98	13.3	3.10	ns
Perimeter *μ*m	72	15.7	62.1	15.20	ns
Roundness	0.515	0.054	0.545	0.05	ns
Tumor volume mm^3^	8245.8	189920.0	2817.9	4305.9	ns

GH	*n* 8		*n* 2		
Density/1.0 mm^2^	51	27.1	59.4	15.10	ns
Area %	5.59	6.56	5.98	3.43	ns
Diameter *μ*m	10.8	2.8	10.8	2.10	ns
Perimeter *μ*m	52.1	18.9	56.8	12.50	ns
Roundness	0.557	0.070	0.473	0.033	ns
Tumor volume mm^3^	3842.1	5570.5	500.0	282.8	ns

ACTH	*n* 0		*n* 3		
Density/1.0 mm^2^	nd		24.2	13.30	nd
Area %	nd		2.72	0.76	nd
Diameter *μ*m	nd		13.5	4.70	nd
Perimeter *μ*m	nd		66.5	20.70	nd
Roundness	nd		0.481	0.057	nd
Tumor volume mm^3^	nd		2264.8	1976.50	nd

TSH	*n* 1		*n* 0		
Density/1.0 mm^2^	51	27.1	nd		nd
Area %	5.59	6.56	nd		nd
Diameter *μ*m	10.8	2.8	nd		nd
Perimeter *μ*m	52.1	18.9	nd		nd
Roundness	0.557	0.070	nd		nd
Tumor volume mm^3^	346.5	nd	nd		nd

**Table 2:** 

mRNA expression	Parameters	*r*	Statistics
VEGF165/actin	Density	0.220	ns
	Area	0.301	ns
	Diameter	0.143	ns
	Perimeter	0.260	ns
	Roundness	−0.026	ns
	Tumor volume	0.110	ns

VEGF121/actin	Density	0.169	ns
	Area	0.292	ns
	Diameter	0.175	ns
	Perimeter	0.218	ns
	Roundness	0.056	ns
	Tumor volume	0.270	ns

**Table 3:** Morphometric analysis of vascular architecture between adenoma function.

	NF		PRL		GH		ACTH		TSH	
	mean	SD	mean	SD	mean	SD	mean	SD	mean	
All cases	*n* 24		*n* 13		*n* 10		*n* 3		*n* 1	
Density/1.0 mm^2^	56.6	33.5	46.8	25.7	52.7	24.6	24.2	13.3	83	
Area %	6.24	4.24	7.59	4.55	5.66	5.90	2.72	0.76	6.27	
Diameter *μ*m	12.1	2.4	14.1^*^	3.0	10.8	2.6	13.5	4.7	9.58	^*^ *P* < 0.05 to GH
Perimeter *μ*m	55.7	14.2	67.5	15.7	53.0	17.3	66.5	20.7	47.5	
Roundness	0.57	0.06	0.529	0.053	0.54	0.072	0.481	0.057	0.471	

VEGF-positive case	*n* 11		*n* 7		*n* 8		*n* 0		*n* 1	
Density/1.0 mm^2^	63.8	36.7	50.3	25.9	51.0	27.1	nd		83	
Area %	7.13	5.37	9.19	5.18	5.59	6.56	nd		6.27	
Diameter *μ*m	11.9	2.7	14.9^*^	3.0	10.8	2.8	nd		9.58	^*^ *P* < 0.05 to GH
Perimeter *μ*m	56.4	17.4	72.0	15.7	52.1	18.9	nd		47.5	
Roundness	0.564	0.032	0.515	0.0543	0.557	0.0699	nd		0.471	

Macroadenoma case	*n* 24		*n* 10		*n* 8		*n* 2		*n* 0	
Density/1.0 mm^2^	56.6	33.3	44.3	24.1	49.2	24.9	16.7	23.3	nd	
Area %	6.2	4.2	8.4	4.6	5.7	6.60	3.1	0.32	nd	
Diameter *μ*m	12.1	2.4	15.2^**^	2.4	11.1	2.8	16.2	0.2	nd	^**^ *P* < 0.01 to NF, GH
Perimeter *μ*m	55.7	14.2	72.7^*^	12.9	53.3	19.4	78.3	4.7	nd	^*^ *P* < 0.05 to NF, GH
Roundness	0.571	0.064	0.527	0.060	0.558	0.069	0.503	0.060	nd	
